# 6-Fluoro-2-(4-meth­oxy­phen­yl)imidazo[2,1-*b*][1,3]benzothia­zole

**DOI:** 10.1107/S1600536811046666

**Published:** 2011-11-12

**Authors:** Hoong-Kun Fun, Madhukar Hemamalini, K. Umesha, B. K. Sarojini, B. Narayana

**Affiliations:** a X-ray Crystallography Unit, School of Physics, Universiti Sains Malaysia, 11800 USM, Penang, Malaysia; b Department of Chemistry, P. A. College of Engineering, Nadupadavu, Mangalore 574 153, India; c Department of Chemistry, Mangalore University, Mangalagangotri, Mangalore 574 199, India

## Abstract

The asymmetric unit of the title compound, C_16_H_11_FN_2_OS, comprises two independent mol­ecules in which the benzothia­zole rings are essentially planar, with maximum deviations of 0.038 (2) and 0.045 (3) Å. The central benzothia­zole ring makes dihedral angles of 4.87 (13) and 0.64 (12)° and 4.04 (12) and 3.67 (12)° with the two terminal phenyl rings in the two independent mol­ecules. In the crystal, mol­ecules are connected *via* weak inter­molecular C—H⋯O hydrogen bonds forming supra­molecular chains along the *c* axis.

## Related literature

For details and applications of benzothia­zoles, see: Yaseen *et al.* (2006 ▶); Kini *et al.* (2007 ▶); Munirajasekhar *et al.* (2011 ▶); Gurupadayya *et al.* (2008 ▶); Mittal *et al.* (2007 ▶); Bowyer *et al.* (2007 ▶); Pozas *et al.* (2005 ▶); Rana *et al.* (2008 ▶); Saha *et al.* (2000 ▶); Katritzky & Rees (1984 ▶). For bond-length data, see: Allen *et al.* (1987 ▶). For a related structure, see: Fun *et al.* (2011 ▶).
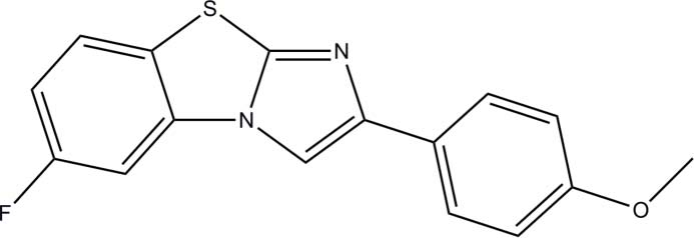


## Experimental

### 

#### Crystal data

C_16_H_11_FN_2_OS
*M* *_r_* = 298.33
Monoclinic, 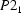

*a* = 7.6120 (13) Å
*b* = 13.883 (2) Å
*c* = 13.049 (2) Å
β = 105.117 (3)°
*V* = 1331.3 (4) Å^3^
*Z* = 4
Mo *K*α radiation
μ = 0.25 mm^−1^
*T* = 296 K
0.31 × 0.30 × 0.13 mm

#### Data collection

Bruker APEXII DUO CCD area-detector diffractometer
Absorption correction: multi-scan (*SADABS*; Bruker, 2009 ▶) *T*_min_ = 0.926, *T*_max_ = 0.967
20506 measured reflections
7656 independent reflections
4268 reflections with *I* > 2σ(*I*)
*R*_int_ = 0.051

#### Refinement

*R*[*F* ^2^ > 2σ(*F* ^2^)] = 0.051
*wR*(*F* ^2^) = 0.116
*S* = 1.00
7656 reflections
381 parameters
1 restraint
H-atom parameters constrained
Δρ_max_ = 0.24 e Å^−3^
Δρ_min_ = −0.25 e Å^−3^
Absolute structure: Flack (1983 ▶), 3649 Friedel pairs
Flack parameter: 0.00 (8)

### 

Data collection: *APEX2* (Bruker, 2009 ▶); cell refinement: *SAINT* (Bruker, 2009 ▶); data reduction: *SAINT*; program(s) used to solve structure: *SHELXTL* (Sheldrick, 2008 ▶); program(s) used to refine structure: *SHELXTL*; molecular graphics: *SHELXTL*; software used to prepare material for publication: *SHELXTL* and *PLATON* (Spek, 2009 ▶).

## Supplementary Material

Crystal structure: contains datablock(s) global, I. DOI: 10.1107/S1600536811046666/bq2315sup1.cif

Structure factors: contains datablock(s) I. DOI: 10.1107/S1600536811046666/bq2315Isup2.hkl

Supplementary material file. DOI: 10.1107/S1600536811046666/bq2315Isup3.cml

Additional supplementary materials:  crystallographic information; 3D view; checkCIF report

## Figures and Tables

**Table 1:** Hydrogen-bond geometry (Å, °)

*D*—H⋯*A*	*D*—H	H⋯*A*	*D*⋯*A*	*D*—H⋯*A*
C6*A*—H6*AA*⋯O1*A*^i^	0.93	2.53	3.367 (5)	149
C6*B*—H6*BA*⋯O1*B*^i^	0.93	2.52	3.382 (4)	153

Symmetry code: (i) 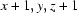.

**Fig. 1.**
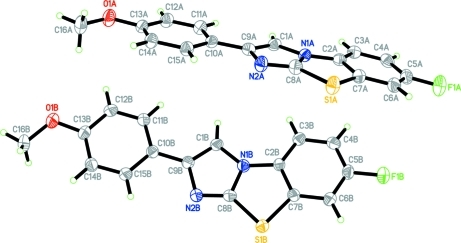
An ORTEP view of the title compound, showing 30% probability displacement ellipsoids.

**Fig. 2.**
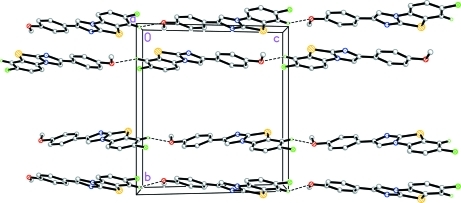
The crystal packing of the title compound (I). H atoms are not involing the hydrogen bond interactions are omitted for clarity.

## supplementary crystallographic information

### Comment

Benzothiazoles are very important bicyclic ring compounds which are of great interest because of their biological activities. The substituted benzothiazole derivatives have emerged as significant components in various diversified therapeutic applications. Literature review has shown that benzothiazoles and their derivatives show considerable properties, including potent inhibition of human immunodeficiency virus type 1 (HIV-1), replication by HIV-1 protease (Yaseen *et al.*, 2006), antitumor (Kini *et al.*, 2007), anthelmintic (Munirajasekhar *et al.*, 2011) analgesic and anti-inflammatory (Gurupadayya *et al.*, 2008), antimalarial (Bowyer *et al.*, 2007), antifungal (Mittal *et al.*, 2007), anticandidous (Pozas *et al.*, 2005) and various CNS activities (Rana *et al.*, 2008). Imidazole has become an important part of many pharmaceuticals. Synthetic imidazoles are present in many fungicides and antifungals (Saha *et al.*, 2000), antiprotozoal and antihypertensive medications. Imidazole is part of the theophylline molecule, found in tea leaves and coffee beans, that stimulates the central nervous system. It is present in the anticancer medication mercaptopurine, which combats leukemia by interfering with DNA activities (Katritzky & Rees, 1984).  The structure of a related compound  7-Chloro-3-phenylbenzo[4,5]thiazolo-[2,3-c][1,2,4] triazole has been reported by Fun *et al.*, 2011.

In continuation of our research investigation, a new fused imidazo[2,1-b]thiazole derivative was prepared by the reaction of flurobenzothiazole  amine with 4-methoxy phenacyl bromide under microwave irradiation at 130 °C and its crystal structure is reported here.

The asymmetric unit of the title compound consists of two crystallographically independent molecules, (A & B), as shown in Fig. 1. The bond lengths and angles of molecules A and B agree with each other and are within normal ranges (Allen *et al.*, 1987).  The benzothiazole units are essentially planar with maximum deviations of  0.038 (2) Å for atom N1A (molecule A) and 0.045 (3) Å  for atom C7B (molecule B).  The dihedral angles that the central benzothiazole (S1/N1,N2/C1,C2,C7–C9) ring makes with the two terminal phenyl (C2–C7/C10–C15) rings in the two independent molecules are 4.87 (13)° : 0.64 (12)° for molecule A and 4.04 (12)° : 3.67 (12)° for molecule B.

In the crystal structure (Fig. 2), the molecules are connected *via* weak intermolecular C—H···O hydrogen bonds forming one-dimensional supramolecular chains along the *c*-axis.

### Experimental

A mixture of 5-fluorobenzothiazole amine  (0.5 g, 2.99 mmol) and 2-bromo-1- (4-methoxyphenyl)ethanone (0.75 g, 3.29 mmol) in ethanol (10 ml) were irradiated with microwave at 130 °C for 45 min. The solvent was evaporated under reduced pressure and the residue was dissolved in dichloromethane (50 ml), washed with 10% sodium bicarbonate (2 X 50 ml), water (50 ml) and finally with saturated brine solution (2 X 50 ml). The organic layer was dried using anhydrous sodium sulphate and then evaporated under vacuum. The crude material was recrystallized by using dichloromethane and methanol to afford the title compound (0.7 g, 78 %) as colorless crystals. mp:450.9–451.9 K (Solvent of Crystallization: 1:1 dichloromethane : methanol).

### Refinement

All hydrogen atoms were positioned geometrically [C–H = 0.93 or 0.96 Å] and were refined using a riding model, with *U*_iso_(H) = 1.2 *U*_eq_(C). A rotating group model was applied to the methyl groups. 3649 Friedel pairs were used to determine the absolute configuration.

### Crystal data

**Table:**

C_16_H_11_FN_2_OS	*F*(000) = 616
*M**_r_* = 298.33	*D*_x_ = 1.488 Mg m^−^^3^
Monoclinic, *P*2_1_	Mo *K*α radiation, λ = 0.71073 Å
Hall symbol: P 2yb	Cell parameters from 3518 reflections
*a* = 7.6120 (13) Å	θ = 2.2–23.5°
*b* = 13.883 (2) Å	µ = 0.25 mm^−^^1^
*c* = 13.049 (2) Å	*T* = 296 K
β = 105.117 (3)°	Block, colourless
*V* = 1331.3 (4) Å^3^	0.31 × 0.30 × 0.13 mm
*Z* = 4	

### Data collection

**Table:**

Bruker APEXII DUO CCD area-detector diffractometer	7656 independent reflections
Radiation source: fine-focus sealed tube	4268 reflections with *I* > 2σ(*I*)
graphite	*R*_int_ = 0.051
φ and ω scans	θ_max_ = 30.0°, θ_min_ = 2.2°
Absorption correction: multi-scan (*SADABS*; Bruker, 2009)	*h* = −8→10
*T*_min_ = 0.926, *T*_max_ = 0.967	*k* = −19→19
20506 measured reflections	*l* = −18→18

### Refinement

**Table:**

Refinement on *F*^2^	Secondary atom site location: difference Fourier map
Least-squares matrix: full	Hydrogen site location: inferred from neighbouring sites
*R*[*F*^2^ > 2σ(*F*^2^)] = 0.051	H-atom parameters constrained
*wR*(*F*^2^) = 0.116	*w* = 1/[σ^2^(*F*_o_^2^) + (0.0433*P*)^2^ + 0.0628*P*] where *P* = (*F*_o_^2^ + 2*F*_c_^2^)/3
*S* = 1.00	(Δ/σ)_max_ = 0.001
7656 reflections	Δρ_max_ = 0.24 e Å^−^^3^
381 parameters	Δρ_min_ = −0.25 e Å^−^^3^
1 restraint	Absolute structure: Flack (1983), 3649 Friedel pairs
Primary atom site location: structure-invariant direct methods	Flack parameter: 0.00 (8)

### Special details

**Table:**

Geometry. All s.u.'s (except the s.u. in the dihedral angle between two l.s. planes) are estimated using the full covariance matrix. The cell s.u.'s are taken into account individually in the estimation of s.u.'s in distances, angles and torsion angles; correlations between s.u.'s in cell parameters are only used when they are defined by crystal symmetry. An approximate (isotropic) treatment of cell s.u.'s is used for estimating s.u.'s involving l.s. planes.
Refinement. Refinement of F^2^ against ALL reflections. The weighted R-factor wR and goodness of fit S are based on F^2^, conventional R-factors R are based on F, with F set to zero for negative F^2^. The threshold expression of F^2^ > 2σ(F^2^) is used only for calculating R-factors(gt) etc. and is not relevant to the choice of reflections for refinement. R-factors based on F^2^ are statistically about twice as large as those based on F, and R- factors based on ALL data will be even larger.

### Fractional atomic coordinates and isotropic or equivalent isotropic displacement parameters (Å^2^)

**Table:**

	*x*	*y*	*z*	*U*_iso_*/*U*_eq_	
S1A	0.85944 (11)	1.02159 (6)	0.83180 (6)	0.0574 (2)	
F1A	1.5287 (3)	0.92386 (17)	0.98451 (18)	0.0787 (7)	
O1A	0.1100 (4)	0.95521 (17)	0.15032 (18)	0.0642 (7)	
N1A	0.9233 (4)	0.96545 (17)	0.65606 (19)	0.0406 (6)	
N2A	0.6279 (4)	1.00305 (19)	0.62596 (19)	0.0499 (7)	
C1A	0.8431 (5)	0.9457 (2)	0.5494 (2)	0.0447 (8)	
H1AA	0.8999	0.9213	0.4999	0.054*	
C2A	1.0919 (5)	0.9560 (2)	0.7307 (3)	0.0416 (8)	
C3A	1.2520 (5)	0.9211 (2)	0.7145 (3)	0.0462 (8)	
H3AA	1.2599	0.9045	0.6468	0.055*	
C4A	1.4000 (5)	0.9111 (2)	0.8010 (3)	0.0517 (9)	
H4AA	1.5099	0.8876	0.7927	0.062*	
C5A	1.3830 (5)	0.9365 (2)	0.9004 (3)	0.0529 (9)	
C6A	1.2256 (5)	0.9728 (3)	0.9189 (3)	0.0513 (9)	
H6AA	1.2195	0.9902	0.9867	0.062*	
C7A	1.0774 (5)	0.9822 (2)	0.8322 (2)	0.0448 (7)	
C8A	0.7857 (5)	0.9987 (2)	0.6965 (3)	0.0475 (8)	
C9A	0.6656 (5)	0.9695 (2)	0.5327 (3)	0.0442 (8)	
C10A	0.5183 (4)	0.9645 (2)	0.4339 (2)	0.0419 (7)	
C11A	0.5515 (5)	0.9338 (2)	0.3389 (3)	0.0512 (9)	
H11A	0.6680	0.9147	0.3376	0.061*	
C12A	0.4119 (5)	0.9315 (2)	0.2468 (3)	0.0535 (9)	
H12A	0.4354	0.9107	0.1840	0.064*	
C13A	0.2379 (5)	0.9599 (2)	0.2466 (2)	0.0469 (8)	
C14A	0.2027 (5)	0.9902 (2)	0.3400 (2)	0.0489 (8)	
H14A	0.0862	1.0094	0.3410	0.059*	
C15A	0.3434 (5)	0.9915 (2)	0.4323 (2)	0.0485 (8)	
H15A	0.3189	1.0113	0.4952	0.058*	
C16A	−0.0712 (5)	0.9810 (3)	0.1471 (3)	0.0736 (11)	
H16A	−0.1439	0.9797	0.0749	0.110*	
H16B	−0.0730	1.0446	0.1755	0.110*	
H16C	−0.1196	0.9361	0.1886	0.110*	
S1B	0.44615 (11)	0.65085 (6)	0.87504 (6)	0.0551 (2)	
F1B	1.1185 (3)	0.74352 (15)	1.02571 (15)	0.0727 (6)	
O1B	−0.3004 (4)	0.72079 (18)	0.19408 (19)	0.0642 (7)	
N1B	0.5109 (4)	0.70099 (17)	0.6977 (2)	0.0420 (7)	
N2B	0.2174 (4)	0.6637 (2)	0.6678 (2)	0.0471 (7)	
C1B	0.4345 (5)	0.7183 (2)	0.5919 (2)	0.0449 (8)	
H1BA	0.4932	0.7411	0.5425	0.054*	
C2B	0.6794 (4)	0.71310 (19)	0.7725 (2)	0.0379 (7)	
C3B	0.8426 (5)	0.7428 (2)	0.7558 (3)	0.0450 (8)	
H3BA	0.8528	0.7565	0.6878	0.054*	
C4B	0.9899 (5)	0.7517 (2)	0.8425 (3)	0.0496 (8)	
H4BA	1.1021	0.7708	0.8336	0.060*	
C5B	0.9712 (5)	0.7326 (2)	0.9415 (3)	0.0500 (9)	
C6B	0.8146 (5)	0.7013 (2)	0.9615 (2)	0.0496 (8)	
H6BA	0.8072	0.6879	1.0301	0.060*	
C7B	0.6651 (4)	0.6903 (2)	0.8739 (2)	0.0427 (8)	
C8B	0.3730 (4)	0.6691 (2)	0.7379 (2)	0.0444 (7)	
C9B	0.2543 (5)	0.6953 (2)	0.5740 (2)	0.0417 (8)	
C10B	0.1091 (5)	0.7020 (2)	0.4758 (3)	0.0423 (8)	
C11B	0.1394 (5)	0.7359 (2)	0.3813 (2)	0.0506 (8)	
H11B	0.2560	0.7550	0.3802	0.061*	
C12B	0.0013 (5)	0.7417 (3)	0.2897 (3)	0.0554 (9)	
H12B	0.0248	0.7652	0.2279	0.066*	
C13B	−0.1737 (5)	0.7124 (2)	0.2892 (2)	0.0472 (8)	
C14B	−0.2083 (5)	0.6788 (2)	0.3807 (3)	0.0505 (9)	
H14B	−0.3251	0.6595	0.3811	0.061*	
C15B	−0.0671 (5)	0.6739 (2)	0.4732 (2)	0.0489 (8)	
H15B	−0.0915	0.6512	0.5351	0.059*	
C16B	−0.4794 (5)	0.6850 (3)	0.1868 (3)	0.0671 (10)	
H16D	−0.5526	0.6914	0.1151	0.101*	
H16E	−0.5333	0.7213	0.2334	0.101*	
H16F	−0.4724	0.6184	0.2071	0.101*	

### Atomic displacement parameters (Å^2^)

**Table:**

	*U*^11^	*U*^22^	*U*^33^	*U*^12^	*U*^13^	*U*^23^
S1A	0.0490 (6)	0.0787 (6)	0.0442 (5)	0.0094 (4)	0.0113 (4)	−0.0105 (4)
F1A	0.0579 (17)	0.1000 (16)	0.0651 (15)	0.0116 (13)	−0.0074 (12)	−0.0116 (12)
O1A	0.0589 (18)	0.0840 (17)	0.0428 (13)	0.0008 (14)	0.0007 (12)	−0.0052 (12)
N1A	0.0385 (18)	0.0436 (14)	0.0396 (15)	0.0029 (12)	0.0104 (12)	0.0004 (12)
N2A	0.0422 (17)	0.0633 (18)	0.0431 (14)	0.0056 (14)	0.0093 (12)	−0.0042 (12)
C1A	0.053 (2)	0.0441 (17)	0.0376 (17)	−0.0003 (14)	0.0128 (15)	0.0002 (12)
C2A	0.045 (2)	0.0360 (16)	0.0452 (18)	0.0008 (15)	0.0144 (15)	0.0016 (13)
C3A	0.050 (2)	0.0409 (16)	0.0503 (19)	0.0022 (15)	0.0175 (17)	−0.0004 (14)
C4A	0.041 (2)	0.0457 (18)	0.068 (3)	0.0004 (16)	0.0143 (19)	−0.0006 (16)
C5A	0.047 (2)	0.053 (2)	0.053 (2)	−0.0049 (16)	0.0036 (17)	−0.0041 (15)
C6A	0.052 (2)	0.055 (2)	0.0446 (19)	−0.0062 (17)	0.0102 (16)	−0.0063 (16)
C7A	0.049 (2)	0.0423 (16)	0.0439 (18)	−0.0001 (15)	0.0130 (15)	−0.0045 (14)
C8A	0.043 (2)	0.054 (2)	0.0478 (19)	0.0048 (16)	0.0155 (15)	−0.0044 (15)
C9A	0.047 (2)	0.0408 (16)	0.0464 (19)	−0.0011 (15)	0.0158 (17)	0.0000 (14)
C10A	0.046 (2)	0.0383 (15)	0.0397 (17)	−0.0018 (14)	0.0077 (14)	0.0010 (13)
C11A	0.050 (2)	0.056 (2)	0.048 (2)	0.0050 (16)	0.0141 (17)	0.0003 (16)
C12A	0.058 (3)	0.064 (2)	0.0387 (18)	0.0004 (17)	0.0125 (17)	−0.0032 (15)
C13A	0.049 (2)	0.0462 (16)	0.0408 (18)	−0.0026 (16)	0.0035 (16)	0.0037 (14)
C14A	0.045 (2)	0.0543 (19)	0.0470 (18)	0.0061 (15)	0.0106 (15)	−0.0025 (14)
C15A	0.052 (2)	0.0525 (19)	0.0405 (18)	−0.0007 (15)	0.0112 (16)	−0.0036 (14)
C16A	0.052 (3)	0.094 (3)	0.064 (2)	0.005 (2)	−0.0045 (18)	0.001 (2)
S1B	0.0432 (5)	0.0785 (6)	0.0425 (4)	−0.0067 (4)	0.0093 (4)	0.0115 (4)
F1B	0.0498 (13)	0.0932 (14)	0.0636 (13)	−0.0094 (11)	−0.0058 (10)	0.0097 (12)
O1B	0.0554 (18)	0.0845 (18)	0.0456 (13)	−0.0021 (14)	0.0009 (12)	0.0108 (13)
N1B	0.0400 (18)	0.0464 (15)	0.0398 (15)	−0.0022 (12)	0.0106 (13)	−0.0020 (12)
N2B	0.0417 (17)	0.0549 (16)	0.0433 (14)	−0.0053 (14)	0.0085 (12)	0.0012 (13)
C1B	0.051 (2)	0.0484 (18)	0.0355 (16)	−0.0038 (15)	0.0121 (15)	0.0004 (13)
C2B	0.0339 (19)	0.0384 (15)	0.0403 (16)	0.0028 (13)	0.0077 (13)	−0.0004 (12)
C3B	0.044 (2)	0.0478 (17)	0.0433 (18)	0.0049 (15)	0.0117 (16)	0.0020 (15)
C4B	0.045 (2)	0.0444 (17)	0.062 (2)	−0.0003 (14)	0.0171 (17)	0.0073 (15)
C5B	0.041 (2)	0.0504 (18)	0.051 (2)	0.0024 (16)	−0.0016 (16)	0.0024 (16)
C6B	0.047 (2)	0.0557 (18)	0.0439 (17)	0.0006 (15)	0.0078 (15)	0.0057 (14)
C7B	0.038 (2)	0.0452 (17)	0.0450 (18)	0.0004 (14)	0.0106 (15)	0.0011 (14)
C8B	0.045 (2)	0.0483 (17)	0.0424 (17)	−0.0028 (15)	0.0157 (15)	0.0026 (14)
C9B	0.045 (2)	0.0432 (16)	0.0373 (17)	−0.0023 (14)	0.0109 (15)	−0.0033 (13)
C10B	0.044 (2)	0.0404 (16)	0.0413 (17)	−0.0024 (14)	0.0083 (15)	−0.0021 (13)
C11B	0.046 (2)	0.0609 (19)	0.0445 (18)	−0.0049 (16)	0.0112 (16)	0.0048 (16)
C12B	0.059 (3)	0.065 (2)	0.0424 (19)	−0.0071 (19)	0.0131 (17)	0.0091 (18)
C13B	0.052 (2)	0.0453 (17)	0.0399 (17)	0.0036 (15)	0.0037 (15)	0.0005 (13)
C14B	0.041 (2)	0.061 (2)	0.049 (2)	−0.0031 (16)	0.0096 (16)	−0.0005 (16)
C15B	0.051 (2)	0.0530 (18)	0.0424 (17)	−0.0059 (15)	0.0106 (15)	0.0026 (14)
C16B	0.048 (2)	0.081 (3)	0.060 (2)	0.0003 (19)	−0.0078 (17)	−0.0053 (18)

### Geometric parameters (Å, °)

**Table:**

S1A—C8A	1.737 (4)	S1B—C8B	1.748 (3)
S1A—C7A	1.746 (3)	S1B—C7B	1.758 (3)
F1A—C5A	1.353 (4)	F1B—C5B	1.359 (3)
O1A—C13A	1.376 (4)	O1B—C13B	1.364 (3)
O1A—C16A	1.415 (4)	O1B—C16B	1.430 (4)
N1A—C8A	1.369 (4)	N1B—C8B	1.364 (4)
N1A—C1A	1.394 (4)	N1B—C1B	1.373 (4)
N1A—C2A	1.400 (4)	N1B—C2B	1.405 (3)
N2A—C8A	1.311 (4)	N2B—C8B	1.296 (4)
N2A—C9A	1.401 (4)	N2B—C9B	1.396 (4)
C1A—C9A	1.352 (5)	C1B—C9B	1.368 (4)
C1A—H1AA	0.9300	C1B—H1BA	0.9300
C2A—C3A	1.378 (5)	C2B—C3B	1.379 (4)
C2A—C7A	1.405 (4)	C2B—C7B	1.392 (4)
C3A—C4A	1.379 (4)	C3B—C4B	1.375 (4)
C3A—H3AA	0.9300	C3B—H3BA	0.9300
C4A—C5A	1.382 (5)	C4B—C5B	1.362 (5)
C4A—H4AA	0.9300	C4B—H4BA	0.9300
C5A—C6A	1.378 (5)	C5B—C6B	1.357 (5)
C6A—C7A	1.380 (4)	C6B—C7B	1.396 (4)
C6A—H6AA	0.9300	C6B—H6BA	0.9300
C9A—C10A	1.473 (4)	C9B—C10B	1.461 (4)
C10A—C15A	1.378 (4)	C10B—C15B	1.389 (5)
C10A—C11A	1.394 (4)	C10B—C11B	1.394 (4)
C11A—C12A	1.382 (4)	C11B—C12B	1.372 (4)
C11A—H11A	0.9300	C11B—H11B	0.9300
C12A—C13A	1.381 (5)	C12B—C13B	1.391 (5)
C12A—H12A	0.9300	C12B—H12B	0.9300
C13A—C14A	1.379 (4)	C13B—C14B	1.370 (4)
C14A—C15A	1.388 (4)	C14B—C15B	1.393 (4)
C14A—H14A	0.9300	C14B—H14B	0.9300
C15A—H15A	0.9300	C15B—H15B	0.9300
C16A—H16A	0.9600	C16B—H16D	0.9600
C16A—H16B	0.9600	C16B—H16E	0.9600
C16A—H16C	0.9600	C16B—H16F	0.9600
			
C8A—S1A—C7A	90.01 (16)	C8B—S1B—C7B	89.75 (16)
C13A—O1A—C16A	117.9 (3)	C13B—O1B—C16B	117.8 (3)
C8A—N1A—C1A	105.7 (3)	C8B—N1B—C1B	106.1 (3)
C8A—N1A—C2A	114.7 (3)	C8B—N1B—C2B	115.2 (2)
C1A—N1A—C2A	139.4 (3)	C1B—N1B—C2B	138.4 (3)
C8A—N2A—C9A	103.7 (3)	C8B—N2B—C9B	104.2 (3)
C9A—C1A—N1A	105.8 (3)	C9B—C1B—N1B	105.9 (3)
C9A—C1A—H1AA	127.1	C9B—C1B—H1BA	127.1
N1A—C1A—H1AA	127.1	N1B—C1B—H1BA	127.1
C3A—C2A—N1A	127.8 (3)	C3B—C2B—C7B	121.0 (3)
C3A—C2A—C7A	121.7 (3)	C3B—C2B—N1B	128.5 (3)
N1A—C2A—C7A	110.4 (3)	C7B—C2B—N1B	110.4 (3)
C2A—C3A—C4A	118.5 (3)	C4B—C3B—C2B	118.1 (3)
C2A—C3A—H3AA	120.8	C4B—C3B—H3BA	120.9
C4A—C3A—H3AA	120.8	C2B—C3B—H3BA	120.9
C3A—C4A—C5A	119.1 (3)	C5B—C4B—C3B	119.9 (3)
C3A—C4A—H4AA	120.5	C5B—C4B—H4BA	120.1
C5A—C4A—H4AA	120.5	C3B—C4B—H4BA	120.1
F1A—C5A—C6A	118.2 (3)	C6B—C5B—F1B	117.5 (3)
F1A—C5A—C4A	118.0 (3)	C6B—C5B—C4B	124.0 (3)
C6A—C5A—C4A	123.8 (3)	F1B—C5B—C4B	118.5 (3)
C5A—C6A—C7A	117.0 (3)	C5B—C6B—C7B	116.5 (3)
C5A—C6A—H6AA	121.5	C5B—C6B—H6BA	121.7
C7A—C6A—H6AA	121.5	C7B—C6B—H6BA	121.7
C6A—C7A—C2A	120.0 (3)	C2B—C7B—C6B	120.3 (3)
C6A—C7A—S1A	127.2 (2)	C2B—C7B—S1B	112.9 (2)
C2A—C7A—S1A	112.7 (2)	C6B—C7B—S1B	126.8 (2)
N2A—C8A—N1A	113.5 (3)	N2B—C8B—N1B	113.6 (3)
N2A—C8A—S1A	134.4 (2)	N2B—C8B—S1B	134.6 (2)
N1A—C8A—S1A	112.1 (3)	N1B—C8B—S1B	111.7 (2)
C1A—C9A—N2A	111.3 (3)	C1B—C9B—N2B	110.2 (3)
C1A—C9A—C10A	129.0 (3)	C1B—C9B—C10B	129.1 (3)
N2A—C9A—C10A	119.6 (3)	N2B—C9B—C10B	120.7 (3)
C15A—C10A—C11A	117.9 (3)	C15B—C10B—C11B	117.1 (3)
C15A—C10A—C9A	120.8 (3)	C15B—C10B—C9B	120.5 (3)
C11A—C10A—C9A	121.3 (3)	C11B—C10B—C9B	122.4 (3)
C12A—C11A—C10A	120.2 (3)	C12B—C11B—C10B	121.6 (3)
C12A—C11A—H11A	119.9	C12B—C11B—H11B	119.2
C10A—C11A—H11A	119.9	C10B—C11B—H11B	119.2
C13A—C12A—C11A	121.0 (3)	C11B—C12B—C13B	120.2 (3)
C13A—C12A—H12A	119.5	C11B—C12B—H12B	119.9
C11A—C12A—H12A	119.5	C13B—C12B—H12B	119.9
O1A—C13A—C14A	124.5 (3)	O1B—C13B—C14B	124.8 (3)
O1A—C13A—C12A	116.0 (3)	O1B—C13B—C12B	115.4 (3)
C14A—C13A—C12A	119.5 (3)	C14B—C13B—C12B	119.8 (3)
C13A—C14A—C15A	119.1 (3)	C13B—C14B—C15B	119.4 (3)
C13A—C14A—H14A	120.4	C13B—C14B—H14B	120.3
C15A—C14A—H14A	120.4	C15B—C14B—H14B	120.3
C10A—C15A—C14A	122.2 (3)	C10B—C15B—C14B	122.0 (3)
C10A—C15A—H15A	118.9	C10B—C15B—H15B	119.0
C14A—C15A—H15A	118.9	C14B—C15B—H15B	119.0
O1A—C16A—H16A	109.5	O1B—C16B—H16D	109.5
O1A—C16A—H16B	109.5	O1B—C16B—H16E	109.5
H16A—C16A—H16B	109.5	H16D—C16B—H16E	109.5
O1A—C16A—H16C	109.5	O1B—C16B—H16F	109.5
H16A—C16A—H16C	109.5	H16D—C16B—H16F	109.5
H16B—C16A—H16C	109.5	H16E—C16B—H16F	109.5
			
C8A—N1A—C1A—C9A	0.9 (3)	C8B—N1B—C1B—C9B	−0.4 (3)
C2A—N1A—C1A—C9A	176.0 (3)	C2B—N1B—C1B—C9B	−174.7 (3)
C8A—N1A—C2A—C3A	177.2 (3)	C8B—N1B—C2B—C3B	179.1 (3)
C1A—N1A—C2A—C3A	2.4 (5)	C1B—N1B—C2B—C3B	−6.9 (5)
C8A—N1A—C2A—C7A	1.0 (3)	C8B—N1B—C2B—C7B	−0.9 (3)
C1A—N1A—C2A—C7A	−173.8 (3)	C1B—N1B—C2B—C7B	173.1 (3)
N1A—C2A—C3A—C4A	−175.2 (3)	C7B—C2B—C3B—C4B	−1.6 (4)
C7A—C2A—C3A—C4A	0.6 (4)	N1B—C2B—C3B—C4B	178.4 (3)
C2A—C3A—C4A—C5A	−0.1 (4)	C2B—C3B—C4B—C5B	−0.9 (4)
C3A—C4A—C5A—F1A	178.4 (3)	C3B—C4B—C5B—C6B	2.3 (5)
C3A—C4A—C5A—C6A	−0.8 (5)	C3B—C4B—C5B—F1B	−179.0 (3)
F1A—C5A—C6A—C7A	−178.1 (3)	F1B—C5B—C6B—C7B	−179.8 (3)
C4A—C5A—C6A—C7A	1.1 (5)	C4B—C5B—C6B—C7B	−1.1 (5)
C5A—C6A—C7A—C2A	−0.6 (4)	C3B—C2B—C7B—C6B	2.8 (4)
C5A—C6A—C7A—S1A	177.5 (3)	N1B—C2B—C7B—C6B	−177.2 (2)
C3A—C2A—C7A—C6A	−0.3 (4)	C3B—C2B—C7B—S1B	−178.2 (2)
N1A—C2A—C7A—C6A	176.2 (3)	N1B—C2B—C7B—S1B	1.8 (3)
C3A—C2A—C7A—S1A	−178.6 (2)	C5B—C6B—C7B—C2B	−1.4 (4)
N1A—C2A—C7A—S1A	−2.1 (3)	C5B—C6B—C7B—S1B	179.7 (3)
C8A—S1A—C7A—C6A	−176.1 (3)	C8B—S1B—C7B—C2B	−1.7 (2)
C8A—S1A—C7A—C2A	2.1 (2)	C8B—S1B—C7B—C6B	177.1 (3)
C9A—N2A—C8A—N1A	0.4 (3)	C9B—N2B—C8B—N1B	−0.7 (3)
C9A—N2A—C8A—S1A	−176.8 (3)	C9B—N2B—C8B—S1B	175.4 (3)
C1A—N1A—C8A—N2A	−0.8 (3)	C1B—N1B—C8B—N2B	0.7 (3)
C2A—N1A—C8A—N2A	−177.3 (2)	C2B—N1B—C8B—N2B	176.5 (2)
C1A—N1A—C8A—S1A	177.0 (2)	C1B—N1B—C8B—S1B	−176.3 (2)
C2A—N1A—C8A—S1A	0.5 (3)	C2B—N1B—C8B—S1B	−0.4 (3)
C7A—S1A—C8A—N2A	175.8 (3)	C7B—S1B—C8B—N2B	−174.9 (3)
C7A—S1A—C8A—N1A	−1.4 (2)	C7B—S1B—C8B—N1B	1.2 (2)
N1A—C1A—C9A—N2A	−0.7 (3)	N1B—C1B—C9B—N2B	0.0 (3)
N1A—C1A—C9A—C10A	179.1 (3)	N1B—C1B—C9B—C10B	178.6 (3)
C8A—N2A—C9A—C1A	0.2 (3)	C8B—N2B—C9B—C1B	0.4 (3)
C8A—N2A—C9A—C10A	−179.6 (3)	C8B—N2B—C9B—C10B	−178.3 (2)
C1A—C9A—C10A—C15A	179.6 (3)	C1B—C9B—C10B—C15B	179.4 (3)
N2A—C9A—C10A—C15A	−0.6 (4)	N2B—C9B—C10B—C15B	−2.2 (4)
C1A—C9A—C10A—C11A	−1.4 (5)	C1B—C9B—C10B—C11B	−0.4 (5)
N2A—C9A—C10A—C11A	178.4 (3)	N2B—C9B—C10B—C11B	178.1 (3)
C15A—C10A—C11A—C12A	0.4 (4)	C15B—C10B—C11B—C12B	0.3 (5)
C9A—C10A—C11A—C12A	−178.7 (3)	C9B—C10B—C11B—C12B	180.0 (3)
C10A—C11A—C12A—C13A	0.3 (5)	C10B—C11B—C12B—C13B	−0.7 (5)
C16A—O1A—C13A—C14A	−1.6 (5)	C16B—O1B—C13B—C14B	−5.1 (4)
C16A—O1A—C13A—C12A	178.3 (3)	C16B—O1B—C13B—C12B	175.4 (3)
C11A—C12A—C13A—O1A	179.6 (3)	C11B—C12B—C13B—O1B	−179.7 (3)
C11A—C12A—C13A—C14A	−0.5 (5)	C11B—C12B—C13B—C14B	0.7 (5)
O1A—C13A—C14A—C15A	179.9 (3)	O1B—C13B—C14B—C15B	−179.8 (3)
C12A—C13A—C14A—C15A	0.0 (5)	C12B—C13B—C14B—C15B	−0.3 (5)
C11A—C10A—C15A—C14A	−0.9 (4)	C11B—C10B—C15B—C14B	0.2 (4)
C9A—C10A—C15A—C14A	178.2 (3)	C9B—C10B—C15B—C14B	−179.6 (3)
C13A—C14A—C15A—C10A	0.7 (4)	C13B—C14B—C15B—C10B	−0.1 (5)

### Hydrogen-bond geometry (Å, °)

**Table:**

*D*—H···*A*	*D*—H	H···*A*	*D*···*A*	*D*—H···*A*
C6A—H6AA···O1A^i^	0.93	2.53	3.367 (5)	149.
C6B—H6BA···O1B^i^	0.93	2.52	3.382 (4)	153.

Symmetry codes: (i) *x*+1, *y*, *z*+1.
